# Sex/Gender-Differences in the Health Effects of Environmental Noise Exposure on Hypertension and Ischemic Heart Disease—A Systematic Review

**DOI:** 10.3390/ijerph18189856

**Published:** 2021-09-18

**Authors:** Sarah Rompel, Alexandra Schneider, Annette Peters, Ute Kraus, on behalf of the INGER Study Group

**Affiliations:** Institute of Epidemiology, Helmholtz Zentrum München, Ingolstädter Landstraße 1, 85764 Neuherberg, Germany; alexandra.schneider@helmholtz-muenchen.de (A.S.); peters@helmholtz-muenchen.de (A.P.); ute.kraus@helmholtz-muenchen.de (U.K.)

**Keywords:** gender, sex, cardiovascular health, hypertension, blood pressure, ischemic heart disease, myocardial infarction, environmental noise

## Abstract

Previous studies have demonstrated cardiovascular health effects of environmental noise exposure, partly showing different effect estimates for males and females. This cannot be explained by biological differences between males and females alone. It is assumed that health outcomes and exposure patterns also depend on gender, determined by social, economic, and cultural factors in society. This systematic review evaluated the current state of how sex/gender is integrated in studies on environmental noise associated with hypertension, blood pressure, and ischemic heart diseases. A systematic literature search was conducted in three different databases, identifying thirty studies published between 1 January 2000 and 2 February 2020. Effects varied, with no consistent findings for both males and females. All studies used a binary operationalization of sex/gender, assuming static differences between males and females. The differentiation between biological and social dimensions of sex/gender was not present in any of the studies and the terms “sex” and “gender” were used interchangeably. However, biological and social dimensions of sex/gender were unconsciously taken up in the discussion of the results. Integrating sex/gender-theoretical concepts into future studies offers great potential to increase the validity of research findings, thus making them more useful for prevention efforts, health promotion, and health care.

## 1. Introduction

Health impacts related to the exposure of environmental noise are a growing issue concerning the general public as well as policy makers in Europe [1]. The World Health Organization (WHO) identified environmental noise as the second most dangerous environmental threat to health after air pollution [1,2]. According to the last report of the European Environment Agency (EEA), published in December 2020, more than 100 million Europeans are exposed to noise levels higher than WHO recommendations [3].

Apart from auditory effects such as hearing loss or impairment, there is evidence for several non-auditory adverse health outcomes, including cardiovascular diseases, associated with long-term exposure to environmental noise [4]. The EEA stated that long-term noise exposure contributes to 48,000 new cases of heart disease in Europe every year [3]. Several pathophysiological pathways might play a role in cardiovascular morbidity induced by environmental noise. The most studied pathway is described by the noise reaction model. It is based on a chronic stress reaction and involves a direct response through perturbation of the autonomic nervous system (fight/flight reaction), as well as an indirect endocrine response mediated by the hypothalamo–pituitary–adrenal (HPA) axis (defeat reaction) [5,6]. The HPA axis has been found to follow sex-specific response patterns and is influenced by the menstrual cycle and hormone status [7,8], which could be one reason why epidemiological studies partly find different noise effects for males and females. However, biological differences between males and females cannot adequately explain health, disease, and exposure patterns alone. These patterns can also depend on gender-related factors determined by cultural, economic, and political conditions in society [9,10]. Several sex/gender-theoretical concepts can be applied in health sciences. Sex is a biological construct and is based on biological characteristics enabling sexual reproduction determined by secondary sex characteristics, gonads, and sex chromosomes. Sex is not unambiguous and is categorized on the basis of social convention. Gender is understood as a social construct. It refers to culture-bound conventions, roles, and behaviors, as well as relations between and among gendered groups. Within and between societies, gender roles, gender relations, and biological expressions of gender vary, typically driven by societal divisions based on power and authority (e.g., class, race/ethnicity, nationality, religion) [11]. The concept of intersectionality assumes heterogeneity within gendered groups and highlights multiple intersecting systems of power, such as gender, class, ethnicity, (dis)ability, sexuality, age, etc., that define an individual. When interpreting these group heterogeneities, it must be noted that intersections and the place of gender within those intersections are “context dependent” on the broader social orders, power relations, and processes of discrimination [12]. Embodiment is a concept that describes the way societal and ecological circumstances affect an individual on a molecular and physiological level over the life course [13].

In keeping with Springer et al. [14], in this systematic review, the term sex/gender is used in order to emphasize that biological and social dimensions are entangled and should not be considered in isolation. Health research is increasingly recognizing that both sex and gender needs to be integrated into future studies. Taking sex/gender-theoretical concepts into account may contribute to improve the validity and relevance of research findings and thus create a stronger evidence base for preventive measures, health promotion, and health care provision. In environment-related health research, however, the biological sex with its dichotomous categories “males” and “females” is focused on, if at all, and gender is still largely neglected [11,12,15,16]. Accordingly, a review carried out for the development of the noise guidelines for the European region [17] and its recent update on the association between environmental noise exposure and the cardiovascular system [18,19] did not address possible differences in effects between males and females.

There is a need for an overview of the current state of how sex/gender-aspects are taken into account in the investigation of the association of environmental noise and cardiovascular health. The aim of the present work is to fill this gap, and in addition, to evaluate if current literature shows sex/gender differences for cardiovascular health effects by noise on the basis of the definitions used in the respective studies.

## 2. Materials and Methods

This systematic review was conducted according to the Preferred Reporting Items for Systematic Reviews and Meta-Analyses (PRISMA) [20]. The PRISMA 2020 Checklist is attached in the Supplementary Materials.

### 2.1. Search Strategy

The aim of the systematic search was to identify epidemiological studies that had analyzed the association between environmental noise and hypertension (HT), blood pressure (BP) changes, and ischemic heart disease (IHD). These outcomes were selected because the evidence found for them in the WHO review was the most comprehensive [18]. The search was restricted to long- and short-term studies in which sex/gender-specific analyses had been performed and were published between 1 January 2000 and 2 February 2020 in English or German language. The comprehensive search was executed within the electronic databases Scopus and MEDLINE, with the search templates PubMed and Web of Science. Publications evaluating sex/gender-differences were identified using the terms “sex”, “gender”, “women”, “men”, “females” and “males”. The employed search terms are stated in the Supplementary Materials.

### 2.2. Selection of Studies

The selection of studies was carried out in duplicate and independently by two reviewers (S.R., U.K.), and subsequently discussed. All identified records were screened by both reviewers for title, abstract, and full text, following a structured procedure. In order to include as many studies as possible, one reviewer screened all full texts of eligible studies as well as all reviews for further relevant records (“snowball method”).

### 2.3. Eligibility Criteria

We considered observational studies of any design (e.g., cohort, case-control, cross-sectional, case-crossover, or ecological). Experimental studies, reviews, guides, and handbooks were not included. With regard to the source of noise exposure, we included road, aircraft, and railway traffic, or community as a whole. Since a variety of methods of HT assessment are used, e.g., blood pressure measurements, self-report in a questionnaire, and/or medication intake, we chose not to set any restrictions based on the method of HT assessment. If a study reported an International Statistical Classification of Diseases and Related Health Problems (ICD)-10 code of I20-I25 or ICD-9 code of 410–414, the outcome was included as IHD in this review. For studies that reported an ICD-10 code of I20-I23, I21-I22 or I21 (ICD-9 code 410), the outcome was included as myocardial infarction (MI). The study population was restricted to non-pregnant adults. A sex/gender-specific analysis (e.g., stratification or interaction analyses) was imperative for the inclusion of a study in the systematic review. Studies considering only one sex/gender group were not taken into account.

### 2.4. Data Extraction and Risk of Bias Assessment

The following data were extracted from all studies meeting the inclusion criteria: data on study characteristics (e.g., study type, study location, study period), data on the outcome (frequency measure, e.g., incidence, prevalence, mortality, ICD-10/9-classification, assessment method), data on population characteristics (study population, population size, age), data on exposure (source, assessment, noise indicator, descriptive parameters), data on statistical methods, confounders used for model adjustment, and results, as well as information on the integration of sex/gender (wording, rationale, conceptualization, operationalization, discussion points) according to the evaluation framework used previously by Bolte et al. [15]. We contacted authors for additional data or clarification whenever necessary.

Risk of bias rating was independently carried out by two reviewers (S.R., U.K.) for each outcome, using the risk of bias assessment instrument for systematic reviews developed by the WHO [21]. For each of the six domains (confounding; selection bias; exposure assessment; outcome measurement; missing data and selective reporting) a low, moderate, or high risk was determined. In accordance with the recommendations, these dimensions were considered separately, and a total score was not calculated. All studies were included in the systematic review, irrespective of the results of the risk of bias rating. Any inconsistencies were solved by discussion. A detailed description can be found in the Supplementary Materials.

## 3. Results

### 3.1. Comprehensive Literature Search

**Figure 1:**
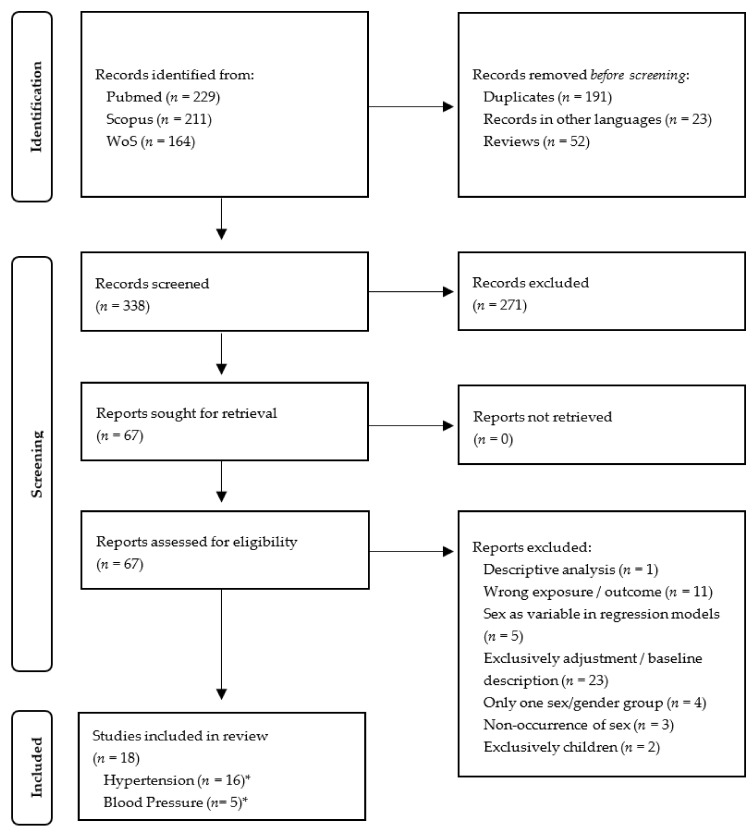
PRISMA Flow Diagram of the selection process of records regarding hypertension and blood pressure-changes. * Three studies investigated both outcomes hypertension and blood pressure-changes.

**Figure 2:**
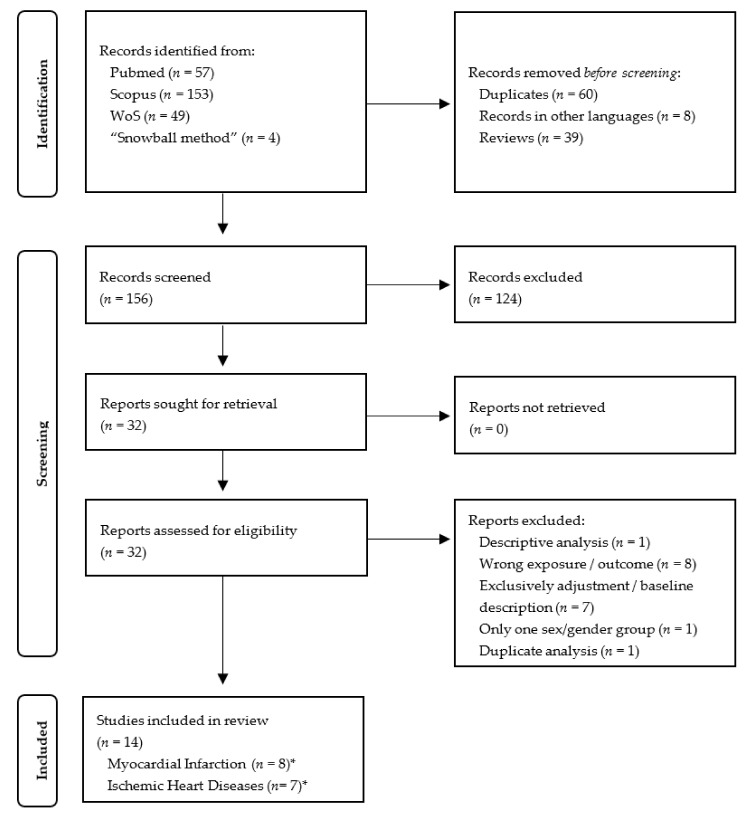
PRISMA Flow Diagram of the selection process of records regarding ischemic heart diseases and myocardial infarction. * One study investigated both outcomes ischemic heart diseases and myocardial infarction.

The selection procedure of all records received through the literature search are illustrated in Figure 1 (HT and BP-changes) and Figure 2 (IHD and MI). A total of 604 records regarding the cardiovascular outcomes HT and BP-changes were identified through database searches (PubMed: 229, Scopus: 211, WoS: 164) (Figure 1). No additional records were identified by the “snowball method”. After exclusion of duplicates and reviews and records published in a language other than English or German, 338 publications were deemed eligible for subsequent selection. A total of 271 records were excluded due to irrelevant titles and abstracts, resulting in 67 articles whose full texts were reviewed in a further step. Then, 49 full-text articles were excluded due to unmet inclusion criteria. Finally, a total of 18 records were eligible for the review, with 13 studies addressing the outcome hypertension, 2 addressing blood pressure changes, and 3 both hypertension and blood pressure changes. All studies included in this review investigated long-term associations of environmental noise with the respective cardiovascular outcomes.

### 3.2. Risk of Bias of the Included Studies

Results from the quality rating are presented in the Supplementary Materials. Overall, studies were rated with low to moderate risk of bias. Confounding was always rated with a low to moderate risk of bias. Only a few studies were rated with a high risk of bias, as critical confounders or antihypertensive medication in studies concerning blood pressure changes had not been taken into account. Selection bias was rated high only in two studies where response rates were low and no information on representativeness was given. Risk of bias due to the exposure assessment was low to moderate in all studies. Risk of bias in outcome measurement was rated low in almost all studies. Only one study was scored with a high risk of bias due to invalid methods of blood pressure measurements. Risk of bias due to missing data was rated low in nearly all of the studies and the risk of bias due to selective reporting was rated low to moderate.

### 3.3. General Study Characteristics

Table 1 gives an overview of all 30 studies included in the systematic review [22,23,24,25,26,27,28,29,30,31,32,33,34,35,36,37,38,39,40,41,42,43,44,45,46,47,48,49,50,51] with population sizes ranging from 308 [46] to 4.6 million participants [41]. Nearly all studies were conducted in European study regions except for two studies conducted in India [23,24], one in North America [38], and one in Korea [46]. Publication years ranged from 2007 to 2019. Fifteen studies conducted cross-sectional analyses [22,23,24,26,27,30,31,32,34,36,37,39,42,43,46] and eleven longitudinal analyses [28,29,33,38,40,41,44,45,47,49,50]. Moreover, three case-control studies [25,48,51] and one ecological study [35] were included in the systematic review. In all studies analyzing the association of environmental noise and hypertension, hypertension was defined as systolic blood pressure (SBP) ≥ 140 mmHg and diastolic blood pressure (DBP) ≥ 90 mmHg. Hypertension was assessed by questionnaire [26,27,36,49], self-reported doctor-diagnosis [33,34], self-reported in combination with the intake of antihypertensive medication [23,30,36,42,43], blood pressure measurements [22,23,30,33,37,43,44,46], or by data obtained from death certificates [25] or patient registers [44,51]. In studies where SBP and/or DBP changes were analyzed, blood pressure was assessed through repeated blood pressure measurements, with protocols differing between studies [31,36,39,43,49]. In one study, measurements were only repeated if SBP was ≥160 mmHg or DBP ≥ 95 mmHg [49] and three studies reported a 5 or 10 min resting time before the first measurement [31,43,49]. Data on ischemic heart diseases were obtained from mortality [25,28,35,48,50] and national patient registers [45,47,50], provincial death registration databases [38], hospital admission records [29,48], hospital discharge registers [48], death certificates [41], national census data [40], or were assessed by self-reported diagnosis by a doctor or qualified practitioner [32,34] in combination with the intake of antihypertensive medication [24]. Environmental noise was either modelled by the Nordic prediction method [28,34,44,45,47,48,49,50] and by help of other national or European algorithms for noise mapping [23,24,25,29,36,37,38,39,40,41,42,51] or assessed through existing noise maps [22,25,32]. In nearly all of the studies, sound pressure levels were assessed as annual mean. In seven studies, no information was provided on the timeframe for which noise exposure was assessed [31,32,34,36,40,46,48].

### 3.4. Conceptualization, Operationalization, Rationale and Discussion of Results

In all 30 studies included in the systematic review, sex/gender was operationalized as a binary construct with the categories males/females or men/women (Table 1). Terminology varied across studies. Eighteen studies used the wording “sex”, five referred to the wording “gender”, and seven used both wordings “sex” and “gender” interchangeably in their publication. One study reported that information on sex/gender of the participants was obtained from death certificates [25], one assessed sex/gender through data from a national population register [27], and another one from data of a national census database [41]. In one study, data on sex/gender were assessed by questionnaire, but no information was given on whether sex/gender was captured with an open question or if specific response categories were provided [32]. No information about the assessment of “sex” and/or “gender” was available in the remaining studies. In 23 studies, no rationale for conducting sex/gender-specific analyses was mentioned. However, seven studies highlighted the importance of their analyses. Reasons mentioned were a different distribution of sex/gender in the study sample and the source population [22], references to previous findings of sex/gender-differences of cardiovascular effects related to noise [23,24,25], no consistent evidence regarding potential sex/gender-differences from previous studies [33,36], or an explanation that “sex was the only variable for which no deviation of the proportional hazard assumption of the Cox models was found” [47]. In all studies, sex/gender-specific analyses were conducted without providing hypotheses regarding the direction of a potential effect.

Seventeen studies evaluated whether sex/gender had an influence on the exposure-outcome association solely by stratification [22,23,24,25,28,30,31,32,35,36,38,39,40,42,46,48,51]. Only 13 studies conducted an interaction analysis [26,27,29,33,34,37,41,43,44,45,47,49,50]. In general, discussion of the results was independent of whether or not differences were found between males and females, and regardless of whether these were determined by interaction analysis or stratification. Four of the thirty studies did not discuss sex/gender-specific results at all [31,32,39,47]. Another four studies only provided a summary of their sex/gender-specific results in the discussion part [22,29,35,48]. Thirteen studies only compared their sex/gender-specific results with results from other studies. If the effects differed between studies, however, the authors did not offer any possible explanations for this [25,26,28,30,34,37,40,41,43,44,49,50,51]. Nine studies made assumptions about possible reasons for the observed differences between sexes/gender [23,24,27,33,36,38,42,45,46]. In general, reasons were mentioned in bullet points and were not elaborated further. The explanations given can be grouped in biological, non-biological, and methodological aspects. Discussed biological aspects potentially explaining higher effects in females included differences in hormones and noise sensitivity [23], use of hormonal contraceptives [27], postmenstrual effects [23], pathophysiological factors in response to noise [24], and differences in the pathogenesis of cardiovascular diseases [33,36]. Additionally, a higher susceptibility to noise-induced stress responses and elevated salivary cortisol levels after noise exposure among females were suggested in two studies [38,45]. Non-biological aspects mentioned as explanations for observed sex/gender-differences included a more stressful marital life and lower employment rates among Indian females [23], different patterns in exposure misclassifications [23,27], and differences in exposure duration [46] as well as a sex/gender-specific “attitude” [24]. However, the meaning of the term “attitude” was not further specified. Other explanations were related to methodological aspects, including unmeasured confounding factors which might be more prevalent among males or females [36], and the occurrence of chance findings [23,27,42]. Only one of the studies that discussed possible explanations found significant differences in the effect estimates for males and females [45].

### 3.5. Sex/Gender Differences in the Association of Environmental Noise and Cardiovascular Diseases

**Figure 3:**
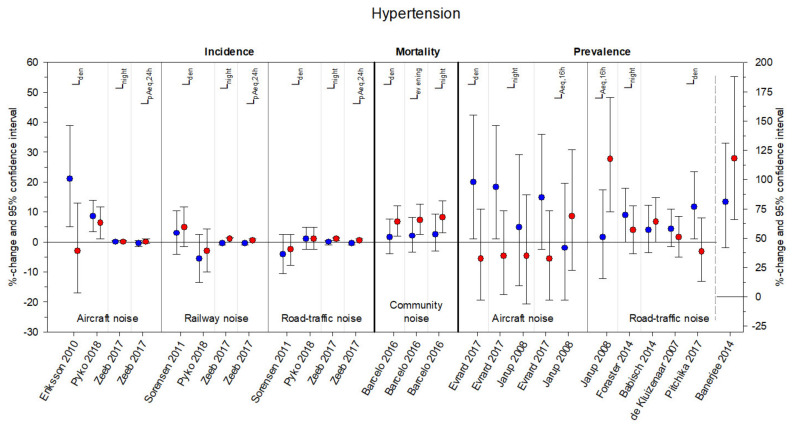
Sex/gender-specific effect estimates from studies with the cardiovascular outcome hypertension. Effect estimates are displayed as percent changes (blue = males, red = females) and 95% confidence intervals per 5 dB(A) increase in noise exposure (aircraft noise, railway noise, road traffic noise, community noise) and grouped according to frequency measure (incidence, mortality, prevalence) and noise indicator (L_den_, L_evening_, L_night_, Lp_Aeq,_ L_Aeq, 16 h_). Please note, some studies provided several estimates for different noise indicators per noise exposure and frequency measure. The scale on the right side of the figure only applies to Banerjee 2014.

**Figure 4:**
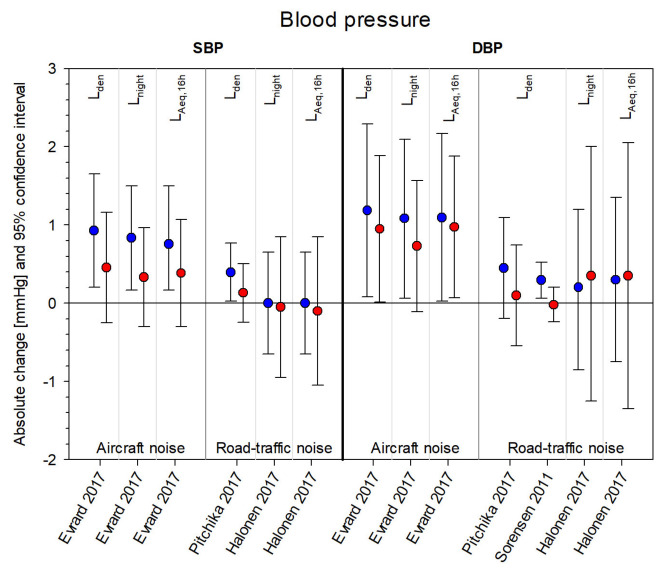
Sex/gender-specific effect estimates from studies with the cardiovascular outcome blood pressure changes. Effect estimates are displayed as absolute changes (mmHg) (blue = males, red = females) and 95% confidence intervals per 5 dB(A) increase in noise exposure (aircraft noise, road traffic noise) and grouped according to systolic and diastolic blood pressure changes (SBP, DBP) and noise indicator (L_den_, L_night_, L_Aeq, 16 h_). Please note, some studies provided several estimates for different noise indicators per noise exposure and frequency measure.

**Figure 5:**
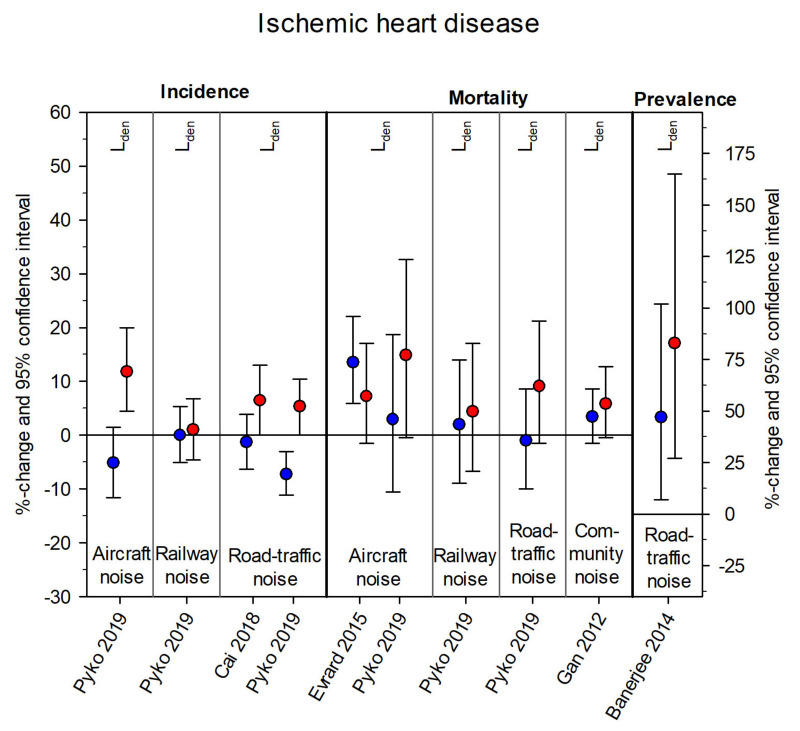
Sex/gender-specific effect estimates from studies with the cardiovascular outcome ischemic heart disease. Effect estimates are displayed as percent changes (blue = males, red = females) and 95% confidence intervals per 5 dB(A) increase in noise exposure (aircraft noise, railway noise, road traffic noise, community noise) and grouped according to frequency measure (incidence, mortality, prevalence) and noise indicator (L_den_). The scale on the right side of the figure only applies to Banerjee 2014.

**Figure 6:**
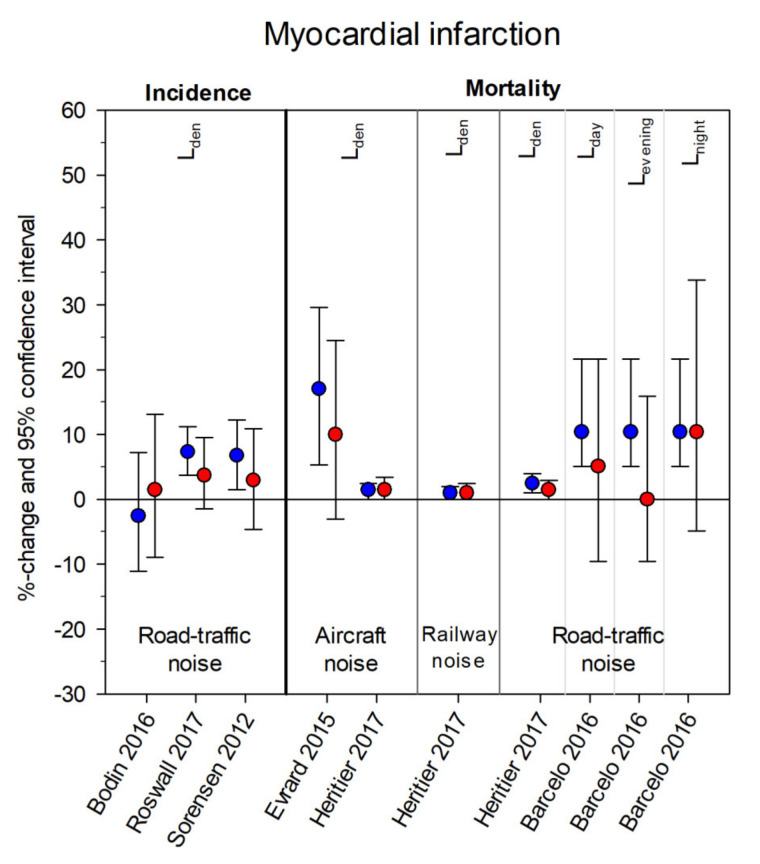
Sex/gender-specific effect estimates from studies with the cardiovascular outcome myocardial infarction. Effect estimates are displayed as percent changes (blue = males, red = females) and 95% confidence intervals per 5 dB(A) increase in noise exposure (aircraft noise, railway noise, road traffic noise) and grouped according to frequency measure (incidence, mortality) and noise indicator (L_den_, L_day_, L_evening_, L_night_). Please note, some studies provided several estimates for different noise indicators per noise exposure and frequency measure.

Figure 3, Figure 4, Figure 5 and Figure 6 summarize the effect estimates for the association between environmental noise and the risk of hypertension, blood pressure changes, ischemic heart disease, and myocardial infarction, respectively. All effect estimates from studies providing continuous estimates for both males (blue) and females (red) were calculated as percent changes and 95% confidence intervals per 5 dB(A) increase in noise exposure. Some studies presented more than one estimate, depending on exposure source, frequency measure, and noise indicator considered.

Supplementary Materials Tables S7–S9 comprise the original effect estimates from all 30 studies including those not reporting any effect sizes for one or both sex/gender groups and those reporting categorical effects, as well as calculated *p*-values for the difference between sex/gender-specific estimates [52]. The majority of estimates were received from studies concerning hypertension: sixteen studies provided 29 effect estimates for females and 28 effect estimates for males. For blood pressure changes, 26 effect estimates (13 each for males and females) were obtained from five studies. Twelve studies concerning ischemic heart disease provided 42 effect estimates (21 each for males and females). The effects were very heterogeneous, with no distinct pattern appearing with respect to noise sources. Only a few significant effects and an even smaller number of significant differences between the sex/gender groups (six for HT none for BP-changes, two for IHD, and none for MI) were found.

## 4. Discussion

Several studies indicate the importance of including sex/gender in scientific research [9,11,16,53,54]. By now, different sex/gender-theoretical concepts exist, all emphasizing that sex/gender is not a binary construct and accordingly, no static differences between male/female, man/woman should be mapped [11,12,55,56]. This review on the association of environmental noise and cardiovascular outcome shows that sex/gender is not given any further meaning in the studies identified, a tendency that has also been recognized for epidemiological studies of other environmental research topics [15,57].

In all 30 studies included in this systematic review, sex/gender-theoretical concepts had not been taken into account in the study design nor in the discussion of sex/gender-specific results. Hence, a dichotomous operationalization using the categories males/females or men/women was applied. Authors used the terms sex, gender, or both interchangeably, suggesting that they did not make a distinction between sex and gender or that they were not aware that there is one at all.

Additionally, most of the studies did not provide any information on how sex/gender of the participants was collected. It remained unclear whether data were obtained from registry information, self-reported during interviews, assigned based on appearance by the interviewer, or self-reported by questionnaire. In the latter case, it is also unclear whether participants had other possibilities to indicate their sex/gender apart from the categories female or male, or woman or man. Those who reported assessment of “sex” by registries, death certificates, or census data might have identified the sex assigned at birth. However, no information was provided regarding the origin of this information, nor what the classification was based on (e.g., genes, genitals, gonads). In the studies that reported assessment of “gender” by questionnaire, it is unclear whether “gender” was actually asked for with an underlying concept, or whether biological sex in terms of the sex assigned at birth was also assessed here.

Uncertainty in how “sex” and/or “gender” was assessed has a major influence on the interpretation of results. It leads to confusion as to whether different effects between males and females are due to differences in their physiological profile, or in social and economic factors. Consequently, no appropriate measures for health care and protection can be derived [11]. Understanding how an interplay of individual, social, and physiological factors produces health disparities for different sex/gender groups could enable the development of appropriate interventions for health protection. Political and social (e.g., gender equality and antidiscrimination programs, access to childcare, etc.) as well as medical interventions (e.g., medication, physical activity) can be considered.

Nevertheless, independently of the used wording, the studies identified in this review did not provide any indication that a concept of gender was included. In a qualitative survey and subsequent workshop to identify challenges associated with the integration of sex and gender in systematic reviews, respondents of a multidisciplinary group of health professionals also noted a tendency for sex to be used as a proxy for gender, as well as an interchangeable usage of the terms [58].

A deeper insight into the mechanisms by which long-term noise can induce cardiovascular diseases is given by Münzel et al. [59]. In the majority of our identified studies, no effect modification was found. One reason for this could be that biological sex actually has very little influence on the association between noise and cardiovascular health. Another more likely explanation could be that dividing the study population in groups on the basis of a sex/gender variable, without an underlying concept, is not appropriate to identify susceptible groups, as differences due to sex/gender variability within the groups might be greater than between them [60,61].

There are no studies providing gender-related analyses in the context of environmental noise exposure to date. The following example illustrates the potential of considering gender-related effects associated with cardiovascular outcomes, even though only aspects relevant for the distinction of males and females were considered. In a study investigating the association of sex and gender with recurrent acute coronary syndrome (ACS) in patients from Canada, gender was included in regression models by a gender-related score [62]. The gender-related score was calculated from gender-related characteristics including gender roles (e.g., childcare), gender identity (e.g., personality traits), gender relationships (e.g., social support), and the institutional gender (e.g., education level, personal income), which were assessed by questionnaire. The study found no differences in ACS recurrence risk by sex independent of gender-related characteristics. However, when the gender-related score was considered in the analysis, participants with characteristics traditionally ascribed to females had a higher risk of recurrent ASC compared to individuals with characteristics traditionally ascribed to males. This confirms the results from a Scottish study on gender roles and quality of life, published in 1990, which found a positive correlation between good health and high masculinity and low femininity irrespective of the biological sex. By then, the authors were already arguing that explanations could not be sought in sex differences alone [63]. This underlines that a definition and operationalization of sex/gender that goes beyond binarism and static differences is needed to detect susceptible population groups.

The authors of the included studies discussed sex/gender-specific differences only marginally and independently of the cardiovascular outcome or the significance of their sex/gender-specific results. Thereby, a distinction between sex and gender was unconsciously taken up as biological, and non-biological aspects were discussed but usually without examining possible explanations. In one study, possible sex/gender-differences were attributed to lower employment rates and more stressful lives among females from a population in India [23]. This thesis could have been tested, as employment status and self-reported mental stress were measured, however, neither was included in the regression models. Differences between employment rate and self-reported mental stress were only tested between the hypertension and the non-hypertension groups. In another study suggesting sex/gender-specific differences in noise sensitivity as a possible explanation for different effects, noise sensitivity was assessed but not included in the regression models [24]. For the remaining discussion points (hormonal differences, post-menstruating effects, usage of hormonal contraceptives, pathophysiological factors towards noise, and differences in the pathogenesis of cardiovascular diseases), no data were available that could have been included in an analysis. For this reason, discussions remained primarily speculative. On the other hand, the studies provide numerous variables, which describe the physiology (e.g., age) or social dimensions of participants’ lives (employment status, marital status, socioeconomic status). However, these variables are generally only used as confounders in the statistical model. Instead, they could have been taken into account in further interaction analyses. Integrating the concept of intersectionality into the models would have even more explanatory potential. However, this would require addressing variables that can explain structural inequalities between different sex/gender groups (e.g., distribution of care work in the family, double burden of paid work and care work, childcare infrastructure, sexist discrimination, exclusion from social power) [60].

Some studies explained higher effect estimates for the associations between environmental noise and cardiovascular outcomes among females by the presence of different patterns in exposure [23,27] or differences in exposure duration [46]. Since differences in the level of exposure do not play a role for the question of effect modification when modeling linear (possibly after transformation) dose–response relationships, the authors probably mean to say that a differential misclassification is present. In general, environmental noise exposure was modelled for participants’ home addresses. No information was available about the noise participants were exposed to outside their residence. If males and females differ in terms of the amount of time spent at home (e.g., due to part- or full-time employment), types of work, as well as other gender-related behaviors, then the degree of exposure misclassification differs between sexes/gender, leading to a systematic bias. Once again, this emphasizes the need to include the social dimensions of gender in environmental health studies. Temporally refined exposure assessment, e.g., by personal noise dosimeters, might be a solution to assess gender-dependent noise exposure. Clougherty et al. proposed the experience sampling method to incorporate gender analyses into environmental studies focusing on air pollution epidemiology [64]. This method from the social sciences could also be applied to noise studies. A comprehensive picture of study participants’ location, activities, and well-being can be obtained by multiple measurements throughout a day, e.g., by using portable devices in combination with diary entries or questionnaires. In addition, these surveys can be continued over several days. Aggregated data of all study participants provide population-specific activity distributions, so exposure differences between sexes/gender could be captured [65,66]. Individual noise assessment would, however, only be applicable in a small study population.

### Strengths and Limitations

Strengths of this systematic review include a transparent methodology that was applied to the entire process and predefined inclusion, exclusion, and quality criteria, which were followed by two independent reviewers. Much effort has also been dedicated to identifying as many studies as possible. Searches were conducted within three different databases, and the snowball method was additionally applied to all studies and all thematically relevant reviews.

A major limitation of the current work was that we could not conduct a meta-analysis. The 30 identified studies encompassed four different sources of noise exposure with three different observed outcomes, which in turn were available as prevalence, incidence, or mortality rate (Figure 3, Figure 4, Figure 5 and Figure 6). Furthermore, six different noise indicators were used, which is why the number of comparable single estimates was too small for pooling. Studies also differed in general study characteristics, e.g., study population and methods to assess the cardiovascular outcome. The high heterogeneity between the studies would have made the interpretation of results of a meta-analysis even more difficult.

## 5. Conclusions

Our review indicates that cardiovascular risk due to environmental noise may differ between males and females. However, identified studies did not properly include sex/gender of the participants in the analysis, or could not do so due to unavailable data. Therefore, it remains unclear if observed effects result from differences in sex-related biology, gender-related factors, or from a combination of both. This strengthens the need to take sex/gender-theoretical concepts into account already in the planning phase of data collection, in order to disentangle sex/gender factors in environmental epidemiology. Only then can adequate conclusions for prevention strategies be derived.

## Figures and Tables

**Table 1:** Characteristics of the included studies.

Reference	Study Type	Study Population	Cardio- Vascular Outcome	Operationalization of Sex/Gender	Assessment of Sex/Gender	Terminology	Rationale for Testing Sex/Gender Differences	Discussion of Sex/Gender-Specific Results
Babisch et al. (2014) [22]	Cross- Sectional	*N* = 1766 (57.3% Female) Berlin, Germany	HT	Binary	Not Reported	Sex, Gender (Inter- changeably)	Different Distribution of Sex/Gender in Study Sample and Source Population	Only Summary of Results
Banerjee et al. (2014a) [23]	Cross- Sectional	*N* = 909 (58.7% Female) Asansol, India	HT	Binary	Not Reported	Gender	Previous Research on Gender Differences in Arterial Hypertension in Relation to Noise Exposure	Comparison with Other Results, Explanations Provided
Banerjee et al. (2014b) [24]	Cross- Sectional	*N* = 909 (58.6% Female) Asansol, India	IHD	Binary	Not Reported	Gender	Previous Research on Gender Differences in Coronary Disease Risk in Relation to Noise Exposure	Comparison with Other Results, Explanations Provided
Barcelo et al. (2016) [25]	Case-Control	*N*_cases HT_ = 4412, *N*_controls HT_ = 4412 *N*_cases MI_ = 6439, *N*_controls MI_ = 6439 Barcelona, Spain	HT, MI	Binary	Death Certificates	Sex	Previous Research on Gender Differences in Adverse Health Effects in Relation to Noise Exposure	Comparison with Other Results
Barregard et al. (2009) [26]	Cross- Sectional	*N* = 1953 (53.0% Female) Lerum, Sweden	HT	Binary	Not Reported	Sex, Gender (Inter- changeably)	Not Reported	Comparison with Other Results
Bluhm et al. (2014) [27]	Cross- Sectional	*N* = 667 (53.5% Female) Municipality 15 km north of Stockholm City, Sweden	HT	Binary	National Population Register	Sex	Not Reported	Comparison with Other Results, Explanations Provided
Bodin et al. (2016) [28]	Cohort	*N* = 12,843 (55.0% Female) Skane, Sweden	MI	Binary	Not Reported	Sex	Not Reported	Comparison with Other Results, Explanations Provided
Cai et al. (2018) [29]	Cohort (HUNT, EPIC Oxford, UK Biobank)	*N* = 355,732 (58.0% Female) Norway; United Kingdom	IHD	Binary	Not Reported	Sex	Not Reported	Only Summary of Results
De Kluizenaar et al. (2007) [30]	Cross- Sectional (PREVENT-Study)	*N* = 7744 (54.7% Female) Groningen, the Netherlands	HT	Binary	Not Reported	Sex, Gender (Inter- changeably)	Not Reported	Comparison with Other Results
Dratva et al. (2012) [31]	Cross- Sectional	*N* = 6450 (51.1% Female) from the Second Survey of the SAPALDIA Cohort Study Switzerland	SBP, DBP	Binary	Not Reported	Sex	Not Reported	–
Dzhambov et al. (2016) [32]	Cross- Sectional	*N* = 513 (64.3% Female) Plovdiv, Bulgaria	IHD	Binary	Questionnaire	Gender	Not Reported	–
Eriksson et al. (2010) [33]	Cohort (SDDP)	*N* = 3902 (63.5% Female) Stockholm County, Sweden	HT	Binary	Not Reported	Sex, Gender (Inter- changeably)	Previous Research on Hypertension in Relation to Aircraft Noise. Uncertainties Regarding Potential Sex/Gender Differences	Comparison with Other Results, Explanations Provided
Eriksson et al. (2012) [34]	Cross- Sectional	*N* = 2493 (55.7% Female) Sweden	HT, IHD	Binary	Not Reported	Sex	Not Reported	Comparison with Other Results
Evrard et al. (2015) [35]	Ecological	*N* = 1.9 million France	IHD, MI	Binary	Not Reported	Gender	Not Reported	Only Summary of Results
Evrard et al. (2017) [36]	Cohort (HYENA-Study)	*N* = 1230 (55.9% Female) France	HT SBP-and DBP-Changes	Binary	Not Reported	Sex, Gender (Inter- changeably)	No Consistent Evidence on Gender Differences in the Risk of Hypertension Related to Aircraft Noise Exposure	Comparison with Other Results, Explanations Provided
Foraster et al. (2014) [37]	Cohort (REGICOR-Study)	*N* = 1929 (54.4% Female) Girona, Spain	HT	Binary	Not Reported	Sex	Not Reported	Comparison with Other Results
Gan et al. (2012) [38]	Cohort	*N* = 445,868 (54.0% Female) Vancouver, Canada	IHD	Binary	Not Reported	Sex	Not Reported	Comparison with Other Results, Explanations Provided
Halonen et al. (2017) [39]	Cross- Sectional (WHII and SABRE Study)	*N* = 4392 (41.0% Female) London, United Kingdom	SBP-and DBP-Changes	Binary	Not Reported	Sex	Not Reported	–
Hertitier et al. (2017) [40]	Cohort (SNC)	*N* = 4,415,206 (47.9% Female) Switzerland	MI	Binary	Not Reported	Sex	Not Reported	Comparison with Other Results
Huss et al. (2010) [41]	Cohort (SNC)	*N* = 4.6 million Switzerland	MI	Binary	National census database	Sex	Not Reported	Comparison with Other Results
Jarup et al. (2008) [42]	Cross- Sectional (HYENA-Study)	*N* = 1076 (50.7% Female) United Kingdom, Germany, the Netherlands, Sweden, Italy, Greece	HT	Binary	Not Reported	Sex	Not Reported	Comparison with Other Results, Explanations Provided
Pitchika et al. (2017) [43]	Cohort (KORA F4)	*N* = 2452 (50.5% Female) Augsburg, Germany	HT SBP- and DBP-Changes	Binary	Not Reported	Sex	Not Reported	Comparison with Other Results
Pyko et al. (2018) [44]	Cohort (SDPP)	*N* = 4854 (59.1% Female) Stockholm county, Sweden	HT	Binary	Not Reported	Sex	Not Reported	Comparison with Other Results
Pyko et al. (2019) [45]	Cohort (SDPP, SIXTY, SALT, SNAC-K)	*N* = 20,012 (57.4% Female) Sweden	IHD	Binary	Not Reported	Sex, Gender (Inter- changeably)	Not Reported	Comparison with Other Results, Explanations Provided
Rhee et al. (2008) [46]	Cross- Sectional	*N*_exposed_ = 308 (58.4% Female), *N*_control_ = 105 (59.0% Female) Korea	HT	Binary	Not Reported	Sex, Gender (Inter- changeably)	Not Reported	Comparison with Other Results, Explanations Provided
Roswall et al. (2017) [47]	Cohort (Diet, Cancer and Health Cohort)	*N* = 50,744 (53.5% Female) Copenhagen or Aarhus, Denmark	MI	Binary	Not Reported	Sex	Sex was the Only Variable for Which no Deviation of the Proportional Hazard Assumption of the Cox Models was Found	-
Selander et al. (2009) [48]	Case-Control (SHEEP)	*N*_cases_ = 1517 (28.6% Female), *N*_controls_ = 2059 (33.7% Female) Stockholm County, Sweden	MI	Binary	Not Reported	Sex	Not Reported	Only Summary of Results
Sørensen et al. (2011) [49]	Cohort (Diet, Cancer and Health Cohort)	*N* = 32,635 Copenhagen or Aarhus, Denmark	HT SBP- and DBP-Changes	Binary	Not Reported	Gender	Not Reported	Comparison with Other Results
Sørensen et al. (2012) [50]	Cohort (Diet, Cancer and Health Cohort)	*N* = 50,614 (52.0% Female) Copenhagen or Aarhus, Denmark	MI	Binary	Not Reported	Sex	Not Reported	Comparison with Other Results
Zeeb et al. (2017) [51]	Case-Control (NORAH)	*N*_cases_ = 137,577 (54.3% Female), *N*_contols_ = 355,591 (54.0% Female) Frankfurt, Germany	HT	Binary	Not Reported	Sex	Not Reported	Comparison with Other Results

HT = hypertension, MI = myocardial infarction, IHD = ischemic heart diseases, SBP = systolic blood pressure, DBP = diastolic blood pressure. Study names: HUNT = Helseundersøkelsen i Nord-Trøndelag, EPIC = European Prospective Investigation into Cancer and Nutrition, PREVEND = Prevention of Renal and Vascular End-Stage Disease, HYENA = Hypertension and Exposure to Noise near Airports, REGICOR = Registre Gironi del Cor; Girona Heart Registry, WHII = Whitehall II, SABRE = Southall and Brent Revisited, SNC = Swiss National Cohort, KORA = Kooperative Gesundheitsforschung in der Region Augsburg, SDPP = Stockholm Diabetes Preventive Program, SALT = Screening Across the Lifespan Twin Study, SNAC-K = Swedish National Study of Aging and Care in Kungsholmen, SHEEP = Stockholm Heart Epidemiology Program, NORAH = noise-related annoyance, cognition, and health.

## Supplementary Materials

The following are available online at https://www.mdpi.com/article/10.3390/ijerph18189856/s1: PRISMA 2020 Checklist, PRISMA 2020 for Abstracts Checklist, Search Strategy, Risk of Bias Assessment Criteria. Rating of the risk of bias of the included studies and reported effect estimates and calculated *p*-values for the difference of sex/gender-specific estimates.
