# Supplementary material for: Sex/Gender-Differences in the Health Effects of Environmental Noise Exposure on Hypertension and Ischemic Heart Disease—A Systematic Review
Source: Int J Environ Res Public Health. 2021 Sep 18;18(18):9856. doi: 10.3390/ijerph18189856 (PMC8465564; doi:10.3390/ijerph18189856)
Supplement: Supplementary file 1 [file ijerph-18-09856-s001.zip › ijerph-1382532-supplementary.pdf]

International Journal of *Environmental Research and Public Health*

MDPI

Systematic Review

Supplementary File: Sex/Gender Differences in the Health Effects of Environmental Noise Exposure on Hypertension and Ischemic Heart Disease – A Systematic Review

1. PRISMA 2020 Checklist

Supplementary Table S1: PRISMA 2020 Checklist [20]

Section and Topic	Item #	Checklist item	Location where item is reported
**TITLE**			
Title	1	Identify the report as a systematic review.	Page 1
**ABSTRACT**			
Abstract	2	See the PRISMA 2020 for Abstracts checklist.	Page 1
**INTRODUCTION**			
Rationale	3	Describe the rationale for the review in the context of existing knowledge.	Page 1-2
Objectives	4	Provide an explicit statement of the objective(s) or question(s) the review addresses.	Page 2
**METHODS**			
Eligibility criteria	5	Specify the inclusion and exclusion criteria for the review and how studies were grouped for the syntheses.	Page 2-3
Information sources	6	Specify all databases, registers, websites, organisations, reference lists and other sources searched or consulted to identify studies. Specify the date when each source was last searched or consulted.	Page 2-3
Search strategy	7	Present the full search strategies for all databases, registers and websites, including any filters and limits used.	Page S5
Selection process	8	Specify the methods used to decide whether a study met the inclusion criteria of the review, including how many reviewers screened each record and each report retrieved, whether they worked independently, and if applicable, details of automation tools used in the process.	Page 3
Data collection process	9	Specify the methods used to collect data from reports, including how many reviewers collected data from each report, whether they worked independently, any processes for obtaining or confirming data from study investigators, and if applicable, details of automation tools used in the process.	Page 3
Data items	10a	List and define all outcomes for which data were sought. Specify whether all results that were compatible with each outcome domain in each study were sought (e.g. for all measures, time points, analyses), and if not, the methods used to decide which results to collect.	Page 2-3
	10b	List and define all other variables for which data were sought (e.g. participant and intervention characteristics, funding sources). Describe any assumptions made about any missing or unclear information.	Page 2-3
Study risk of bias assessment	11	Specify the methods used to assess risk of bias in the included studies, including details of the tool(s) used, how many reviewers assessed each study and whether they worked independently, and if applicable, details of automation tools used in the process.	Page 3, Table S3

**Section and Topic**	**Item #**	**Checklist item**	**Location where item is reported**
Effect measures	12	Specify for each outcome the effect measure(s) (e.g. risk ratio, mean difference) used in the synthesis or presentation of results.	Table 2-4
Synthesis methods	13a	Describe the processes used to decide which studies were eligible for each synthesis (e.g. tabulating the study intervention characteristics and comparing against the planned groups for each synthesis (item #5)).	-
	13b	Describe any methods required to prepare the data for presentation or synthesis, such as handling of missing summary statistics, or data conversions.	-
	13c	Describe any methods used to tabulate or visually display results of individual studies and syntheses.	-
	13d	Describe any methods used to synthesize results and provide a rationale for the choice(s). If meta-analysis was performed, describe the model(s), method(s) to identify the presence and extent of statistical heterogeneity, and software package(s) used.	-
	13e	Describe any methods used to explore possible causes of heterogeneity among study results (e.g. subgroup analysis, meta-regression).	-
	13f	Describe any sensitivity analyses conducted to assess robustness of the synthesized results.	-
Reporting bias assessment	14	Describe any methods used to assess risk of bias due to missing results in a synthesis (arising from reporting biases).	-
Certainty assessment	15	Describe any methods used to assess certainty (or confidence) in the body of evidence for an outcome.	-
**RESULTS**			
Study selection	16a	Describe the results of the search and selection process, from the number of records identified in the search to the number of studies included in the review, ideally using a flow diagram.	Page 4-5
	16b	Cite studies that might appear to meet the inclusion criteria, but which were excluded, and explain why they were excluded.	-
Study characteristics	17	Cite each included study and present its characteristics.	Page 5-7, Table 1
Risk of bias in studies	18	Present assessments of risk of bias for each included study.	Table S4-S6
Results of individual studies	19	For all outcomes, present, for each study: (a) summary statistics for each group (where appropriate) and (b) an effect estimate and its precision (e.g. confidence/credible interval), ideally using structured tables or plots.	Table 2-4
Results of syntheses	20a	For each synthesis, briefly summarise the characteristics and risk of bias among contributing studies.	-
	20b	Present results of all statistical syntheses conducted. If meta-analysis was done, present for each the summary estimate and its precision (e.g. confidence/credible interval) and measures of statistical heterogeneity. If comparing groups, describe the direction of the effect.	-
	20c	Present results of all investigations of possible causes of heterogeneity among study results.	-
	20d	Present results of all sensitivity analyses conducted to assess the robustness of the synthesized results.	-
Reporting biases	21	Present assessments of risk of bias due to missing results (arising from reporting biases) for each synthesis assessed.	-

Section and Topic	Item #	Checklist item	Location where item is reported
Certainty of evidence	22	Present assessments of certainty (or confidence) in the body of evidence for each outcome assessed.	-
**DISCUSSION**			
Discussion	23a	Provide a general interpretation of the results in the context of other evidence.	Page 7-9
	23b	Discuss any limitations of the evidence included in the review.	Page 9
	23c	Discuss any limitations of the review processes used.	Page 9
	23d	Discuss implications of the results for practice, policy, and future research.	Page 9
**OTHER INFORMATION**			
Registration and protocol	24a	Provide registration information for the review, including register name and registration number, or state that the review was not registered.	-
	24b	Indicate where the review protocol can be accessed, or state that a protocol was not prepared.	-
	24c	Describe and explain any amendments to information provided at registration or in the protocol.	-
Support	25	Describe sources of financial or non-financial support for the review, and the role of the funders or sponsors in the review.	Page 9
Competing interests	26	Declare any competing interests of review authors.	Page 10
Availability of data, code and other materials	27	Report which of the following are publicly available and where they can be found: template data collection forms; data extracted from included studies; data used for all analyses; analytic code; any other materials used in the review.	-

Supplementary Table S2: PRISMA 2020 for Abstracts Checklist [20]

Section and Topic	Item #	Checklist item	Reported (Yes/No)
**TITLE**			
Title	1	Identify the report as a systematic review.	Yes
**BACKGROUND**			
Objectives	2	Provide an explicit statement of the main objective(s) or question(s) the review addresses.	Yes
**METHODS**			
Eligibility criteria	3	Specify the inclusion and exclusion criteria for the review.	No

Section and Topic	Item #	Checklist item	Reported (Yes/No)
Information sources	4	Specify the information sources (e.g. databases, registers) used to identify studies and the date when each was last searched.	Yes
Risk of bias	5	Specify the methods used to assess risk of bias in the included studies.	No
Synthesis of results	6	Specify the methods used to present and synthesise results.	No
**RESULTS**			
Included studies	7	Give the total number of included studies and participants and summarise relevant characteristics of studies.	Yes
Synthesis of results	8	Present results for main outcomes, preferably indicating the number of included studies and participants for each. If meta-analysis was done, report the summary estimate and confidence/credible interval. If comparing groups, indicate the direction of the effect (i.e. which group is favoured).	No
**DISCUSSION**			
Limitations of evidence	9	Provide a brief summary of the limitations of the evidence included in the review (e.g. study risk of bias, inconsistency and imprecision).	No
Interpretation	10	Provide a general interpretation of the results and important implications.	Yes
**OTHER**			
Funding	11	Specify the primary source of funding for the review.	No
Registration	12	Provide the register name and registration number.	No

2. Search strategy

2.1 Pubmed

(noise[TI] OR noise*[TI] NOT (rat[TI] OR rats[TI] OR mouse[TI] OR mice[TI])) AND ("ischaemic heart disease"[TIAB] OR IHD[TIAB] OR "ischemic heart disease"[TIAB] OR "ischemic cardiac disease"[TIAB] OR "ischaemic cardiac disease"[TIAB] OR "coronary artery disease"[TIAB] OR CAD[TIAB] OR "coronary heart disease"[TIAB] OR CHD[TIAB] OR "ischemic heart diseases"[TIAB] OR "ischaemic heart diseases"[TIAB] OR "ischemic cardiac diseases"[TIAB] OR "ischaemic cardiac diseases"[TIAB] OR "coronary artery diseases"[TIAB] OR "coronary heart diseases"[TIAB]) AND (sex OR gender OR men OR male* OR women OR female*)

2.2 Web of Sciences

(TI=(noise OR noise*) AND TS=("ischaemic heart disease" OR IHD OR "ischemic heart disease" OR "ischemic cardiac disease" OR "ischaemic cardiac disease" OR "coronary artery disease" OR CAD OR "coronary heart disease" OR CHD OR "ischemic heart diseases" OR "ischaemic heart diseases" OR "ischemic cardiac diseases" OR "ischaemic cardiac diseases" OR "coronary artery diseases" OR "coronary heart diseases") AND TS=(sex OR gender OR men OR male* OR women OR female*) NOT TI=(rat OR rats OR mouse OR mice))

2.3 Scopus

(TITLE(noise)) AND (TITLE-ABS-KEY(cardiovascular OR CVD)) AND (ALL ("ischaemic heart disease" OR IHD OR "ischemic heart disease" OR "ischemic cardiac disease" OR "ischaemic cardiac disease" OR "coronary artery disease" OR CAD OR "coronary heart disease" OR CHD OR "ischemic heart diseases" OR "ischaemic heart diseases" OR "ischemic cardiac diseases" OR "ischaemic cardiac diseases" OR "coronary artery diseases" OR "coronary heart diseases")) AND (ALL(sex OR gender OR men OR women OR male* OR female*)) AND NOT (TITLE (mice OR mouse OR rat* OR occupation*))

3. Risk of Bias Assessment Criteria

Supplementary Table S3: Risk of bias assessment criteria [21]

Domain	Subdomain	Low-risk criteria	Moderate-risk criteria	High-risk criteria
**Confounding**	Were all confounders considered adjusted for in the analysis? (**HT / BP: critical confounders**: age; **other potential confounders:** any SES, BMI, physical activity; smoking; **IHD: critical confounders**: age; smoking **other potential confounders:** any SES, BMI, physical activity)	All critical and other /additional potential confounders adjusted for or with support (e.g. exploratory analysis) of minimal risk due to residual confounding (i.e. there is evidence that this confounder might not lead to severe confounding)	All critical potential confounders but not all other /additional potential confounders adjusted for without support (e.g. exploratory analysis) of minimal risk due to residual confounding	Not all critical potential confounders adjusted for without support (e.g. exploratory analysis) of minimal risk due to residual confounding
	Validity of measuring of confounding factors	Confounders measured with documented valid methods	Not all critical potential confounders were measured with documented valid methods; however, there is evidence that this does not lead to severe confounding or no information about confounder measurement were given	Any critical or other/additional potential confounder not validly assessed and evidence of residual confounding
	Control in analysis (did the authors use an appropriate analysis method (stratification, standardization, adjustment, restriction, matching) or study design (randomization, matching, restriction) that controlled for confounders?)	authors used appropriate analysis methods or study designs that controlled for confounders<br>**BP**: inclusion of anti-hypertensive medication in strata to control for effect-modification	authors used inappropriate methods or designs when adjusting for critical potential confounders; however there is evidence that this does not lead to severe confounding	authors used inappropriate methods or study designs when adjusting for critical and other / additional potential confounders<br>**BP**: no anti-hypertensive medication included in stratified analysis
**Selection Bias**	Selection of participants into the study (includes non-response)	participants in all exposure levels and with all outcomes had equal opportunity to be in the study (randomly selected from population registries or whole population invited and representative for the target population, even if response rate was low (<60%))	participants in all exposure levels did not have equal opportunity to be in the study, but not to the extent that effect estimates were seriously biased (systematic or not randomly selected but representative for the target population, even if response rate was low (<60%) or no information about representativeness were given)	participants in all exposure levels did not have equal opportunity to be in the study, to the extent that effect estimates were seriously biased (systematic or not randomly selected AND not representative for the target population due to response rate less than 60% OR attrition rate higher than 20%)
**Exposure assessment**	methods used for exposure assessment (model, grid)	exposure levels assessed with appropriate methods (use of an appropriate model and precise to geocoded address)	exposure levels assessed with less than appropriate methods (no appropriate model or larger grid) but not to the extent that effect estimates where seriously biased	exposure levels not assessed with appropriate methods (no appropriate model and larger grid) to the extent that effect estimates were seriously biased

	exposure measurement methods comparable across the range of exposure	Measurement methods used are comparable across the range of exposure	Measurement methods vary across the range of exposure; however, there is evidence supporting that the exposure measurement is sufficiently similar that effect estimates are not seriously biased	Measurement methods vary across the range of exposure and differences are not accounted for
	change in exposure status	spatial exposure contrasts did not change throughout the study or time varying exposure was used to account for changes	spatial exposure contrasts did change throughout the study and were not accounted for but effect estimates were not seriously biased (or no information given)	spatial exposure contrasts did change throughout the study and were not accounted for and effect estimates were seriously biased and were different in cases and non-cases
	exposure contrast	Exposure contrast was large compared to the precision of exposure assessment (between-subject variance larger than within subject variance).	Exposure contrast was small relative to the within subject variance but not to the extent that the study is uninformative.	Exposure contrast was so small relative to the within-subject variance that the study is uninformative
**Outcome measurement**	blinding of outcome measurement	outcome measurements were not influenced by knowledge of the exposure	outcome measurements were influenced by knowledge of the exposure; however evidence supports that effect estimates were unlikely biased	outcome detection was related to exposure status and effect estimates are likely biased
	validity of outcome measurements	no systematic errors in the measurement of the outcome or systematic errors were unrelated to the exposure (doctors validated diagnosis, death registries, objectively measured, questionnaire or interview using a known scale or validated assessment method)	minimum systematic errors suspected in the measurement were related to the exposure received	critical systematic errors in the measurement were related to the exposure received
	outcome measurement	Methods of outcome assessment were comparable across exposure groups / centers in multicenter studies.	Methods of outcome assessment were not comparable across exposure groups / centers in multicenters studies; however, evidence supports that outcome detection would not have varied.	Methods of outcome assessment were not comparable across exposure groups / centers in multicenter studies.
**Missing data**	missing data of outcome measures	No missing outcome data or missing data infrequent (<10%) or missing data related to outcome or exposure data imputed using appropriate methods. Attrition rate >20%	Missing data on outcomes not infrequent (≥10%) and rationale for attrition explained in the study; methods have possibly been used to properly account for it. Attrition rate <20% but representative	Evidence of substantial missing outcome data (≥10%), rationale for attrition not explained in the study and methods unlikely to properly account for it. Attrition rate <20%

	missing data of exposures	No missing exposure data or missing data infrequent (<10%) or missing data related to exposure or outcome data imputed using appropriate methods.	Missing data on exposure not infrequent (≥10%) and rationale for attrition explained in the study; methods have possibly been used to properly account for it.	Evidence of substantial missing exposure data (≥10%), rationale for missing data not explained in the study, and/ or the portion of participants and reasons for missing data are dissimilar across exposures/ exposure groups.
**Selective reporting**	authors reported a priori primary and secondary study aims	Effect estimates presented for all hypotheses tested as per aims; reference to published or unpublished study protocol.	Effect estimates presented for some (not all) hypotheses tested as per aims, but evidence suggests that effect estimates unlikely to be seriously biased.	Effect estimates selectively presented for some (not all) hypotheses tested as per aims and effect estimates likely to be seriously biased.

HT = hypertension, BP = blood pressure, SES = socio economic status, BMI = body mass index, IHD = ischemic heart diseases

The subdomain confounding and all criteria were specified in parts following Van Kempen et al. [16].

4. Rating of Risk of Bias of the included studies

Supplementary Table S4: Risk of bias rating of studies concerning the outcome hypertension: overall results of the six domains confounding, selection bias, exposure assessment, outcome measurement, missing data and selective reporting

Reference	Exposure	Confounding	Selection Bias	Exposure assessment	Outcome measurement	Missing data	Selective reporting
**Babisch et al. (2014) [22]**	Road traffic noise	low	moderate	low	low	low	low
**Banerjee et al. (2014a) [23]**	Road traffic noise	moderate	moderate	moderate	low	low	low
**Barcelo et al. (2016) [25]**	Road traffic noise	high	low	moderate	low	low	low
**Barregard et al. (2009) [26]**	Road traffic noise	moderate	low	low	moderate	low	moderate
**Bluhm et al. (2007) [27]**	Road traffic noise	moderate	low	moderate	moderate	low	low
**De Kluizenaar et al. (2007) [30]**	Road traffic noise	moderate	moderate	low	low	moderate	low
**Eriksson et al. (2010) [33]**	Road traffic noise	low	moderate	moderate	moderate	low	moderate
**Eriksson et al. (2012) [34]**	Road traffic noise	moderate	high	low	moderate	low	moderate
**Evrard et al. (2017) [36]**	Aircraft noise	moderate	moderate	moderate	low	low	moderate
**Foraster et al. (2014) [37]**	Road traffic noise	low	moderate	moderate	low	low	moderate
**Jarup et al. (2008) [42]**	Road traffic noise	moderate	moderate	low	low	low	low
	Aircraft noise	moderate	moderate	low	low	low	low

Reference	Exposure	Confounding	Selection Bias	Exposure assessment	Outcome measurement	Missing data	Selective reporting
**Pitchika et al. (2017) [43]**	Road traffic noise	moderate	low	moderate	low	low	low
**Pyko et al. (2018) [44]**	Road traffic noise	moderate	moderate	moderate	low	low	moderate
	Railway noise	moderate	moderate	moderate	low	low	moderate
	Aircraft noise	moderate	moderate	low	low	low	moderate
**Rhee et al. (2008) [46]**	Aircraft noise	moderate	moderate	high	low	low	moderate
**Sørensen et al. (2011) [49]**	Road traffic noise	low	moderate	moderate	low	low	moderate
	Railway noise	low	moderate	low	low	low	moderate
**Zeeb et al. (2017) [51]**	Road traffic noise	moderate	low	moderate	low	low	moderate
	Railway noise	moderate	low	moderate	low	low	moderate
	Aircraft noise	moderate	low	moderate	low	low	moderate

Supplementary Table S5: Risk of bias rating of studies concerning the outcome blood pressure-changes: overall results of the six domains confounding, selection bias, exposure assessment, outcome measurement, missing data and selective reporting

Reference	Exposure	Confounding	Selection Bias	Exposure assessment	Outcome measurement	Missing data	Selective reporting
**Dratva et al. (2012) [31]**	Railway noise	high	low	moderate	low	low	high
**Evrard et al. (2017) [36]**	Aircraft noise	high	moderate	moderate	low	low	moderate
**Halonen et al. (2017) [39]**	Road traffic noise	moderate	low	low	low	low	low
**Pitchika et al. (2017) [43]**	Road traffic noise	moderate	low	moderate	low	low	low
**Sørensen et al. (2011) [49]**	Road traffic noise	low	moderate	moderate	high	low	moderate

36

Supplementary Table 6: Risk of bias rating of studies concerning the outcome ischemic heart disease: overall results of the six domains confounding, selection bias, exposure assessment, outcome measurement, missing data and selective reporting

Reference	Exposure	Confounding	Selection Bias	Exposure assessment	Outcome measurement	Missing data	Selective reporting
**Banerjee et al. (2014b) [24]**	Road traffic noise	moderate	moderate	low	moderate	low	low
**Barcelo et al. (2016) [25]**	Road traffic noise	high	low	moderate	low	low	low
**Bodin et al. (2016) [28]**	Road traffic noise	moderate	low	low	low	low	moderate
**Cai et al. (2018) [29]**	Road traffic noise	moderate	moderate	moderate	low	moderate	low
**Dzhambov et al. (2016) [32]**	Road traffic noise	moderate	high	moderate	moderate	high	moderate
**Eriksson et al. (2012) [34]**	Road traffic noise	moderate	high	low	moderate	low	moderate
**Evrard et al. (2015) [35]**	Aircraft noise	moderate	low	low	low	low	low
	Aircraft noise	moderate	low	low	low	low	low
**Gan et al. (2012) [38]**	Community noise	high	low	moderate	low	low	low
**Heritier et al. (2017) [40]**	Road traffic noise	high	low	low	low	low	low
	Railway noise	high	low	low	low	low	low
	Aircraft noise	high	low	moderate	low	low	low
**Huss et al. (2010) [41]**	Aircraft noise	high	low	moderate	low	low	moderate
**Pyko et al. (2019) [44]**	Road traffic noise	moderate	moderate	moderate	low	low	moderate
	Railway noise	moderate	moderate	moderate	low	low	moderate
	Aircraft noise	moderate	moderate	low	low	low	moderate
	Road traffic noise	moderate	moderate	moderate	low	low	moderate

	Railway noise	moderate	moderate	moderate	low	low	moderate
	Aircraft noise	moderate	moderate	low	low	low	moderate
**Roswall et al. (2017) [47]**	Road traffic noise	moderate	moderate	low	low	low	low
**Selander et al. (2009) [48]**	Road traffic noise	moderate	low	low	low	low	low
**Sørensen et al. (2012) [50]**	Road traffic noise	moderate	moderate	low	low	low	low

Supplementary Table 7: Reported effect estimates from studies with the cardiovascular outcome hypertension and calculated p-value for the difference of sex/gender-specific estimates

Reference	Exposure source	Frequency measure	Noise indicator	Effect measure per increase in noise exposure	Effect in males	Effect in females	p-value[b]
Eriksson et al. (2010) [33]	Aircraft	Incidence	L_{den}	RR (95% CI) per 10 dB(A) increase	**1.21 (1.05-1.39)**	0.97 (0.83-1.13)	**0.038**
Pyko et al. (2018) [44]	Aircraft	Incidence	L_{den}	HR (95% CI) per 10 dB(A) increase	**1.18 (1.07-1.30)**	**1.13 (1.02-1.25)**	0.547
Zeeb et al. (2017) [51]	Aircraft	Incidence	L_{night}	OR (95% CI) per 10 dB(A) increase	1.00 (1.00-1.01)	1.00 (1.00-1.01)	1.000
		Incidence	$Lp_{Aeq, 24 h}$	OR (95% CI) per 10 dB(A) increase	0.99 (0.97-1.01)	1.00 (0.99-1.02)	0.585
Evrard et al. (2017) [36]	Aircraft	Prevalence	L_{den}	OR (95% CI) per 10 dB(A) increase	**1.44 (1.02-2.03)**[a]	0.89 (0.65-1.23)[a]	0.064
		Prevalence	L_{night}	OR (95% CI) per 10 dB(A) increase	**1.40 (1.02-1.93)**[a]	0.91 (0.68-1.22)[a]	0.069
		Prevalence	$L_{Aeq, 16 h}$	OR (95% CI) per 10 dB(A) increase	1.32 (0.95-1.85)[a]	0.89 (0.65-1.22)[a]	0.114
Jarup et al. (2008) [42]	Aircraft	Prevalence	L_{night}	OR (95% CI) per 10 dB(A) increase	1.10 (0.73-1.67)	0.91 (0.63-1.34)	0.527
		Prevalence	$L_{Aeq, 16 h}$	OR (95% CI) per 10 dB(A) increase	0.96 (0.65-1.43)	1.18 (0.82-1.71)	0.466
Rhee et al. (2008) [46]	Aircraft	Prevalence	L_{day}	not reported	not reported	not reported	not reported
Barcelo et al. (2016) [25]	Community	Mortality	L_{day}	OR (95% CI) per 10 dB(A) increase	1.00 (0.99-1.02)	1.01 (1.00-1.02)	0.189
		Mortality	$L_{evening}$	OR (95% CI) per 10 dB(A) increase	1.00 (0.99-1.02)	1.01 (1.01-1.02)	0.189
		Mortality	L_{night}	OR (95% CI) per 10 dB(A) increase	1.01 (0.99-1.02)	**1.02 (1.01-1.03)**	0.168
Pyko et al. (2018) [44]	Railway	Incidence	L_{den}	HR (95% CI) per 10 dB(A) increase	0.89 (0.75-1.05)	0.94 (0.81-1.09)	0.633
Sørensen et al. (2011)	Railway	Incidence	L_{den}	IRR (95% CI) per 5 dB(A)	1.06 (0.92-1.22)	1.10 (0.94-1.25)	0.702

Reference	Exposure source	Frequency measure	Noise indicator	Effect measure per increase in noise exposure	Effect in males	Effect in females	p-value[b]
[49]				increase			
Zeeb et al. (2017) [51]	Railway	Incidence	L_{night}	OR (95% CI) per 10 dB(A) increase	0.99 (0.98-1.00)	1.02 (1.00-1.03)	**0.002**
		Incidence	$Lp_{Aeq, 24 h}$	OR (95% CI) per 10 dB(A) increase	0.99 (0.98-1.00)	1.01 (1.00-1.02)	**0.013**
Pyko et al. (2018) [44]	Road traffic	Incidence	L_{den}	HR (95% CI) per 10 dB(A) increase	0.92 (0.80-1.05)	0.95 (0.85-1.05)	0.713
Sørensen et al. (2011) [49]	Road traffic	Incidence	L_{den}	IRR (95% CI) per 10 dB(A) increase	1.02 (0.95-1.10)	1.02 (0.95-1.10)	1.000
Zeeb et al. (2017) [51]	Road traffic	Incidence	L_{night}	OR (95% CI) per 10 dB(A) increase	1.00 (0.98-1.01)	**1.02 (1.01-1.03)**	**0.003**
		Incidence	$Lp_{Aeq, 24 h}$	OR (95% CI) per 10 dB(A) increase	0.99 (0.98-1.00)	1.01 (1.00-1.02)	**0.011**
Babisch et al. (2014) [22]	Road traffic	Prevalence	L_{den}	OR (95% CI) per 10 dB(A) increase	1.08 (0.93-1.26)	1.14 (1.00-1.32)	0.593
Banerjee et al. (2014a) [23]	Road traffic	Prevalence	L_{den}	OR (95% CI) per 5 dB(A) increase	**1.81 (1.42-2.31)**	**2.18 (1.66-2.88)**	0.337
Barregard et al. (2009) [26]	Road traffic	Prevalence	$L_{Aeq, 24 h}$	POR (95% CI) in 51-55dB noise category	1.60 (0.70-3.90)	1.10 (0.50-2.60)	0.609
		Prevalence	$L_{Aeq, 24 h}$	POR (95% CI) in 56-70dB noise category	**3.80 (1.60-9.00)**	0.90 (0.40-2.30)	0.137
Bluhm et al. (2007) [27]	Road traffic	Prevalence	$L_{Aeq, 24 h}$	OR (95% CI) per 5 dB(A) increase	not reported	**1.71 (1.17-2.50)**	not reported
De Kluizenaar et al. (2007) [30]	Road traffic	Prevalence	L_{den}	OR (95% CI) per 10 dB(A) increase	1.09 (0.97-1.23)	1.03 (0.90-1.18)	0.538
Eriksson et al. (2012) [34]	Road traffic	Prevalence	L_{day}	POR	not reported	not reported	not reported
Foraster et al. (2014) [37]	Road traffic	Prevalence	L_{night}	OR (95% CI) per 5 dB(A) increase	1.09 (1.00-1.18)	1.04 (0.96-1.12)	0.416
Jarup et al. (2008)	Road traffic	Prevalence	$L_{Aeq, 24 h}$	OR (95% CI) per 10 dB(A)	1.03 (0.77-1.38)	**1.63 (1.21-2.20)**	**0.043**

Reference	Exposure source	Frequency measure	Noise indicator	Effect measure per increase in noise exposure	Effect in males	Effect in females	p-value[b]
[42]				increase			
Pitchika et al. (2017) [43]	Road traffic	Prevalence	L_{den}	Percent change (95% CI) per 5 dB(A) increase	**11.73 (1.11-23.47)[a]**	-3.2 (-13.18-7.94)	0.058

[a] effect estimates obtained by request of the corresponding authors
[b] p-value for the difference of the sex/gender-specific estimates calculated using a method proposed by Altman and Bland [52]
Significant effects are indicated as bold values
RR =relative risk; risk ratio, HR = hazard ratio, OR = odds ratio, IRR = incidence rate ratio, POR = prevalence odds ratio, CI = confidence interval

Supplementary Table 8: Reported effect estimates from studies with the cardiovascular outcome systolic and diastolic blood pressure changes (SBP, DBP) and calculated p-value for the difference of sex/gender-specific estimates

Reference	Exposure source	Outcome	Noise indicator	Effect measure per increase in noise exposure	Effect in males	Effect in females	p-value[b]
Evrard et al. (2017) [36]	Aircraft	SBP	L_{night}	absolute change (95% CI) per 10 dB(A) increase	2.17 (0.13-4.19)[a]	1.46 (-0.22-3.13)[a]	0.597
		SBP	L_{den}	absolute change (95% CI) per 10 dB(A) increase	2.37 (0.16-4.59)[a]	1.90 (0.03-3.77)[a]	0.751
		SBP	$L_{Aeq,\ 16\ h}$	absolute change (95% CI) per 10 dB(A) increase	2.19 (0.05-4.34)[a]	1.95 (0.14-3.76)[a]	0.867
		DBP	L_{night}	absolute change (95% CI) per 10 dB(A) increase	1.67 (0.34-3.00)[a]	0.67 (-0.60-1.93)[a]	0.286
		DBP	L_{den}	absolute change (95% CI) per 10 dB(A) increase	1.86 (0.40-3.30)[a]	0.91 (-0.50-2.32)[a]	0.357
		DBP	$L_{Aeq,\ 16\ h}$	absolute change (95% CI) per 10 dB(A) increase	1.51 (0.34-3.00)[a]	0.77 (-0.60-2.14)[a]	0.447
Halonen et al. (2017) [39]	Aircraft	SBP	L_{night}	absolute change (95% CI) per 10 dB(A) increase	0.40 (-1.70-2.40)	0.70 (-2.50-4.00)	0.878
		SBP	$L_{Aeq,\ 16\ h}$	absolute change (95% CI)	0.60 (-1.50-2.70)	0.70 (-2.70-4.10)	0.961

Reference	Exposure source	Outcome	Noise indicator	Effect measure per increase in noise exposure	Effect in males	Effect in females	p-value[b]
				per 10 dB(A) increase			
		DBP	L_{night}	absolute change (95% CI) per 10 dB(A) increase	0.00 (-1.30-1.30)	-0.10 (-1.90-1.70)	0.930
		DBP	$L_{Aeq, 16 h}$	absolute change (95% CI) per 10 dB(A) increase	0.00 (-1.30-1.30)	-0.20 (-2.10-1.70)	0.865
Dratva et al. (2012) [31]	Railway	not reported	not reported	not reported	not reported	not reported	not reported
Pitchika et al. (2017) [43]	Road traffic	SBP	L_{den}	percent change (95% CI) per 5 dB(A) increase	0.36 (-0.16-0.88)	0.08 (-0.44-0.60)	0.451
		DBP	L_{den}	percent change (95% CI) per 5 dB(A) increase	0.51 (0.03-0.99)	0.17 (-0.31-0.65)	0.327
Sørensen et al. (2011) [49]	Road traffic	SBP	L_{den}	absolute change (95% CI) per 10 dB(A) increase	0.59 (0.13-1.05)	-0.04 (-0.48-0.40)	0.052

[a] effect estimates obtained by request of the corresponding authors

[b] p-value for the difference of the sex/gender-specific estimates calculated using a method proposed by Altman and Bland [52]
Significant effects are indicated as bold values
SBP = systolic blood pressure, DBP = diastolic blood pressure, CI = confidence interval

Supplementary Table 9: Reported effect estimates from studies with the cardiovascular outcome ischemic heart disease (IHD) and myocardial infarction (MI) and calculated p-value for the difference of sex/gender-specific estimates

Reference	Exposure source	Frequency measure	Noise indicator	Effect measure per increase in noise exposure	Effect in males	Effect in females	p-value[b]
Evrard et al. (2015) [35]	Aircraft	Mortality IHD	L_{den}	MRR (95% CI) per 10 dB(A) increase	**1.29 (1.12-1.49)**	1.15 (0.97-1.37)	0.314
		Mortality MI	L_{den}	MRR (95% CI) per 10 dB(A) increase	**1.37 (1.11-1.68)**	1.21 (0.94-1.55)	0.453
Heritier et al. (2017) [40]	Aircraft	Mortality MI	L_{den}	HR (95% CI) per 10 dB(A) increase	1.03 (1.00-1.05)	1.03 (1.00-1.07)	0.827
Huss et al. (2010) [41]	Aircraft	Mortality MI	L_{den}	HR (95% CI) >= 60 dB(A)	not reported	not reported	not reported

**Reference**	**Exposure source**	**Frequency measure**	**Noise indicator**	**Effect measure per increase in noise exposure**	**Effect in males**	**Effect in females**	**p-value[b]**
Pyko et al. (2019) [45]	Aircraft	Incidence IHD	L_{den}	HR (95% CI) per 10 dB(A) increase	0.90 (0.78-1.03)	**1.25 (1.09-1.44)**	**0.001**
		Mortality IHD	L_{den}	HR (95% CI) per 10 dB(A) increase	1.06 (0.80-1.41)	1.32 (0.99-1.76)	0.300
Gan et al. (2012) [38]	Community	Mortality IHD	L_{den}	RR (95% CI) per 10 dB(A) increase	1.07 (0.97-1.18)	1.12 (0.99-1.27)	0.576
Heritier et al. (2017) [40]	Railway	Mortality MI	L_{den}	HR (95% CI) per 10 dB(A) increase	1.02 (1.00-1.04)	1.02 (1.00-1.05)	0.616
Pyko et al. (2019) [45]	Railway	Incidence IHD	L_{den}	HR (95% CI) per 10 dB(A) increase	1.00 (0.90-1.11)	1.02 (0.91-1.14)	0.801
		Mortality IHD	L_{den}	HR (95% CI) per 10 dB(A) increase	1.04 (0.83-1.30)	1.09 (0.87-1.37)	0.775
Banerjee et al. (2014b) [24]	Road traffic	Prevalence IHD	L_{den}	OR (95% CI) per 5 dB(A) increase	**1.47 (1.07-2.02)**	**1.83 (1.27-2.65)**	0.400
Barcelo et al. (2016) [25]	Road traffic	Mortality MI	L_{day}	OR (95% CI) per 1 dB(A) increase	**1.02 (1.01-1.04)**	1.01 (0.98-1.04)	0.423
		Mortality MI	$L_{evening}$	OR (95% CI) per 1 dB(A) increase	**1.02 (1.01-1.04)**	1.00 (0.98-1.03)	0.233
		Mortality MI	L_{night}	OR (95% CI) per 1 dB(A) increase	**1.02 (1.01-1.04)**	1.02 (0.99-1.06)	0.881
Bodin et al. (2016) [28]	Road traffic	Incidence MI	L_{den}	IRR (95% CI) per 10 dB(A) increase	0.95 (0.79-1.15)	1.03 (0.83-1.28)	0.586
Cai et al. (2018) [29]	Road traffic	Incidence IHD	L_{den}	HR (95% CI) per 3.9 dB(A) increase	0.99 (0.95-1.03)	1.05 (1.00-1.10)	0.083
Dzhambov et al. (2016) [32]	Road traffic	Prevalence IHD	L_{den}	RR (95% CI) > =65 dB(A)	2.35 (0.64-8.61)[a]	0.61 (0.21-1.78)[a]	0.401
Eriksson et al. (2012) [34]	Road traffic	Prevalence IHD	L_{den}	not reported	not reported	not reported	not reported
Heritier et al. (2017) [40]	Road traffic	Mortality MI	L_{den}	HR (95% CI) per 10 dB(A) increase	**1.05 (1.02-1.08)**	1.03 (1.00-1.06)	0.301

Reference	Exposure source	Frequency measure	Noise indicator	Effect measure per increase in noise exposure	Effect in males	Effect in females	p-value[b]
Pyko et al. (2019) [45]	Road traffic	Incidence IHD	L_{den}	HR (95% CI) per 10 dB(A) increase	0.86 (0.79-0.94)	1.11 (1.00-1.22)	**<0.001**
		Mortality IHD	L_{den}	HR (95% CI) per 10 dB(A) increase	0.98 (0.81-1.18)	1.19 (0.97-1.47)	0.186
Roswall et al. (2017) [47]	Road traffic	Incidence MI	L_{den}	HR (95% CI) per 10.5 dB(A) increase	**1.16 (1.08-1.25)**	1.08 (0.97-1.21)	0.286
Selander et al. (2009) [48]	Road traffic	Prevalence MI	$L_{Aeq, 16 h}$	OR (95% CI) ≥50 dB(A)	not reported	not reported	not reported
Sørensen et al. (2012) [50]	Road traffic	Incidence MI	L_{den}	IRR (95% CI) per 10 dB(A) increase	**1.14 (1.03-1.26)**	1.06 (0.91-1.23)	0.426

[a] effect estimates obtained by request of the corresponding authors
[b] p-value for the difference of the sex/gender-specific estimates calculated using a method proposed by Altman and Bland [52]
Significant effects are indicated as bold values
MRR = mortality risk ratio, RR =relative risk; risk ratio, HR = hazard ratio, OR = odds ratio, IRR = incidence rate ratio, CI = confidence interval
